# Discovery of peptide biomarkers in male *Eupolyphaga sinensis* Walker (Tubiechong) by dimethyl labeling-based quantitative peptidomics analysis

**DOI:** 10.3389/fphar.2026.1799023

**Published:** 2026-07-09

**Authors:** Hui Ge, Xiaozheng Huang, Ruizi Qing, Ping Liu, Haoyu Li, Jiaqi Zhu, Yuanqing Wei, Shuixin Liu, Xun Liu, Shuying Han, Haishan Deng, Rui Liu

**Affiliations:** 1 Jiangsu Collaborative Innovation Center of Chinese Medicinal Resources Industrialization, National and Local Collaborative Engineering Center of Chinese Medicinal Resources Industrialization and Formulae Innovative Medicine, National Administration of Traditional Chinese Medicine Key Laboratory for Chinese Medicine Resources Recycling Utilization, Nanjing University of Chinese Medicine, Nanjing, China; 2 Jiangsu Key Laboratory of Research and Development in Marine Bio-resource Pharmaceutics, Nanjing University of Chinese Medicine, Nanjing, China; 3 Jiangsu Marine Drugs Research and Development Center, Nanjing University of Chinese Medicine, Nanjing, China; 4 School of Pharmacy, Nanjing University of Chinese Medicine, Nanjing, China; 5 Xinxing Tuyuan Specialized Cooperatives of Huangtang Town, Danyang, China; 6 Suzhou Vocational Health College, Suzhou, China; 7 Animal-Derived Chinese Medicine and Functional Peptides International Collaboration Joint Laboratory, Nanjing University of Chinese Medicine, Nanjing, China; 8 State Key Laboratory for Quality Ensurance and Sustainable Use of Dao-di Herbs, Beijing, China

**Keywords:** dimethyl labeling peptidomics, *Eupolyphaga sinensis*, peptide biomarkers, quality control, sex distiction

## Abstract

*Eupolyphaga sinensis* Walker (ES) is traditionally valued for its ability to invigorate blood circulation, resolve blood stasis, eliminate blood clots, and promote the healing of tendons and bones, which has a long-standing history of use and extensive clinical applications. According to the Chinese Pharmacopoeia, only the female insect is approved for medicinal use, yet distinguishing male ES from female ES remains challenging with conventional methods. This work is committed to discover peptide biomarkers to distinguish males from females using dimethyl labeling-based quantitative peptidomics strategy. The analysis revealed significant differences in peptide abundance, leading to the identification of three male ES derived characteristic peptides that could be used to distinguish males from females; these were confirmed and validated using UPLC-MS/MS and MRM mode. These peptide biomarkers could accurately detect the presence of male adulteration in female ES samples across varying mixing ratios as well. These findings provide a practical molecular tool for quality control and standardization of ES medicinal materials.

## Introduction

*Eupolyphaga sinensis* Walker, commonly known as “Tubiechong” (ground beetle), refers to the dried female adult insects belonging to the family Corydiidae. First documented in the “Shennong Bencao Jing” (Shennong’s herbal classic, which is thought to originate in the 3rd century *Anno Domini*), it was classified as a medium-grade medicinal substance and is traditionally valued for its ability to invigorate blood circulation, resolve blood stasis, eliminate blood clots, and promote the healing of tendons and bones. As a significant category of insect-derived animal medicines, *E. sinensis* (ES) has a long-standing history of use and extensive clinical applications. Jiangsu province represents one of its major production regions and is widely recognized as a high-quality entomological medicinal material. Studies have demonstrated that ES contains a variety of bioactive constituents, including proteins, peptides, amino acids, nucleosides, polysaccharides, and alkaloids (Fu et al., 2022; Ni et al., 2024). It exhibits multiple pharmacological activities, such as anti-osteoporotic effects, promotion of bone regeneration, anticoagulant properties, liver protection, and antioxidant activity (Wang et al., 2012; Zhang et al., 2019; Han et al., 2022; Wei et al., 2023; Feng et al., 2024). Its specific mechanisms include enhancing fibrinolysis, improving hemorheological parameters, reducing blood viscosity, stimulating osteoblast proliferation, and inducing the differentiation of bone marrow mesenchymal stem cells. From the 1963 to the 2025 edition, the Chinese Pharmacopoeia has consistently specified that only the female insect of ES is to be used for medicinal purposes, with the male insect excluded. Furthermore, the morphological characteristics described in historical Materia medica literature are consistent with those of the female specimen, supporting this traditional practice (Chinese Pharmacopoeia, 2025).

Currently, the distinction between male and female ES primarily depends on morphological characteristics. Adult males exhibit distinct eclosion features, whereas females lack such traits. However, immature males prior to eclosion are smaller in size and morphologically resemble females, often resulting in misidentification. This challenge is particularly pronounced when ES is processed into powdered form or incorporated into Chinese patent medicines, where conventional macroscopic and microscopic identification techniques are insufficient for accurate sex discrimination (Zhang, 2011; Wu et al., 2024). Therefore, the development of reliable methods for male and female ES identification, especially for detecting the adulteration of female samples with male individuals, has become a crucial aspect of quality control in ES medicinal materials.

In recent years, molecular identification techniques have been increasingly employed for the authentication of insect-derived medicinal materials. For example, using Illumina/Solexa deep sequencing technology, 45 microRNAs (miRNAs) exhibiting significantly differential expression between male and female ES have been identified (Wu et al., 2013). Mitochondrial gene sequences, including COI, Cytb, and 16S rRNA, have also been utilized for species discrimination (Li et al., 2015; Zou et al., 2025). However, due to the absence of comprehensive genomic and transcriptomic data for ES, its sex-determination mechanism and associated genes remain poorly understood. As a result, effective sex-linked DNA molecular markers have not yet been developed, thereby constraining the application of DNA barcoding technologies in this field (Fan et al., 2024).

Currently, characteristic peptide detection technology has demonstrated considerable promise for the authentication of animal-derived traditional Chinese medicines. The 2025 edition of the Chinese Pharmacopoeia has incorporated characteristic peptides as identification or quantitative markers for products including Saigae Tataricae Cornu (Liu et al., 2023), Corii Asini Colla (Cai et al., 2021), Cervi Cornus Colla (Han et al., 2021), and Testudinis Carapacis et Plastri Colla (Yang et al., 2015). In the domain of traditional Chinese medicine formula granules, this technology has also been applied to authenticate insect-based ingredients such as ES, Scolopendra, and Pheretima (Li et al., 2022; He et al., 2023; Hu et al., 2023; Luo et al., 2023). Building upon this foundation, the present study employed a dimethyl labeling-based quantitative peptidomics approach to systematically compare and analyze differential peptides between male and female ES. Furthermore, this study successfully identified male ES derived characteristic peptide markers, which can be utilized to detect adulteration of female-derived materials with male insects, demonstrating effective discrimination across various adulteration ratios. This finding is of substantial significance for enhancing the quality control of ES medicinal materials and advancing related standardization efforts.

## Materials and methods

### Reagents and materials

LC-MS grade formic acid, acetonitrile (ACN) and methanol (MeOH) were purchased from Thermo Fisher Scientific (Waltham, MA, United States). Water was prepared on a Milli-Q Gradient A10 system by Millipore (Schwalbach, Germany) and used for all buffers and other solutions including LC eluents. All other chemicals and reagents were of the highest grade available. Trypsin (sequencing grade) was obtained from Promega (Fitchburg, WI, United States). Peptide biomarkers shown in Table 1 were chemically synthesized by GenScript Corporation (Nanjing, China). Male and female *E. sinensis* were collected from Xinxing Tuyuan Specialized Cooperatives of Huangtang Town, Danyang.

**TABLE 1:** Amino acid sequence information and MRM parameters for peptide biomarkers.

Peptides	Sequences	Precursors	Q1 (*m/z*)	Q3 (*m/z*)	DP	CE
ES-pep-1	EMSWIADTYAK	Glutamate dehydrogenase (P54385)	657.9	781.4	31.62	31.22
				668.2	80.50	39.88
ES-pep-2	IVPIVEPEVLPDGDHDLDR	Fructose-bisphosphate aldolase (P07764)	710.4	639.5	33.47	24.83
				739.6	44.24	33.30
ES-pep-3	TGAIVDVPVGDELLGR	ATP synthase subunit alpha (P35381)	806.1	955.2	71.32	33.92
				343.1	49.93	38.08
ES-pep-R	EAFSLFDKDGDGTITTK	Calmodulin (P62152)	615.6	649.5	89.35	27.08
				823.1	66.36	25.02

DP: decluttering potential; CE: collision energy.

### Sample preparation

**FIGURE 1:**
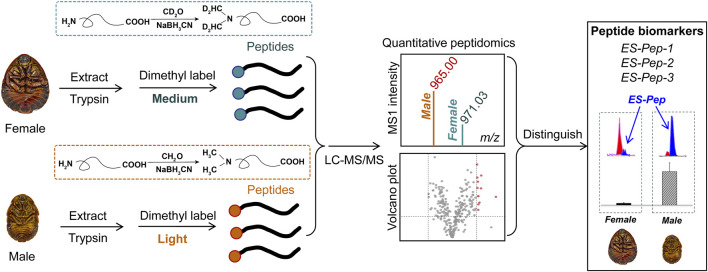
Analytical scheme for peptide biomarkers identification for distinguishing male ES from female ES.

The whole body of ES was crushed, 30 mg of ES powder was taken and lysed by sonication in a solution containing digest buffer (4% SDS, 100 mM Tris pH 8.0). The BCA assay was applied to determine the protein concentration. Then 200 μg protein was incubated for 0.5 h after adding DTT to 10 mM, and then samples were alkylated with 55 mM iodoacetamide (IAA) in the dark for 30 min, and the reaction was quenched by 5 mM DTT. Proteins were precipitated from the soluble extract by addition of 5.5 volumes of 80% acetone overnight and rinsed with 80% acetone, and then dried in air. Dried pellet was resuspended in 8 M urea, 30 mM NaCl, 50 mM Tris (pH = 8). The sample was sonicated in an ice-water bath for 20 min, centrifuged at 14,000 × *g* for 5 min, and the supernatant was collected. After that, the supernatant was diluted with 50 mM Tris solution (pH = 8) to a urea concentration of less than 1 M. Protein digestion was performed with trypsin enzyme at a 50:1 protein/enzyme ratio at 37 °C for 16 h. The digestion was quenched by adding 10% trifluoroacetic acid (TFA) to a pH lower than 3, 35 μL of each tryptic sample was taken and diluted to 100 μL with 0.5 M triethylammonium bicarbonate (TEAB) buffer (Sigma). The peptides from female sources were labeled with light isotope-coded chemicals (HCHO and NaBH_3_CN), while those from male sources were labeled with medium isotope-coded chemicals (DCDO and NaBH_3_CN), as shown in Figure 1. After labeling, equal amounts of light-labeled and medium-labeled samples were mixed and desalted using Waters C18 Sep-Pak cartridge (Massachusetts, United States). Samples were dried under Vacuum Concentrator (Labconco, Kansas City, MO, United States) and redissolved in 0.1% formic acid (FA) prior to nanoLC-MS/MS analysis.

### NanoLC-MS/MS analysis

Dimethyl labeled samples were analyzed using a Dionex Ultimate 3000 nanoLC system (Thermo Scientific) coupled to a Q Exactive Plus quadrupole-Orbitrap mass spectrometer (Thermo Fisher Scientific, San Jose, CA). Two microliters of each sample in 0.1% formic acid was directly injected and separated at a flow rate of 300 nL min^−1^ on a lab-fabricated reverse phase capillary column (75 μm × 15 cm, particle size 1.7 μm, pore size 150 Å). Mobile phase A consisted of water with 0.1% FA, and mobile phase B was composed of ACN with 0.1% FA. Separation was performed using a gradient elution of 3%–30% mobile phase B over 90 min.

Parameters of nano spray were set as follows: capillary temperature, 350 °C; spray voltage, 2.2 kV. A data-dependent acquisition under positive mode alternated between one MS scan followed by MS/MS scans for the top 20 most abundant precursor ions, normalized collision energy was set as 30. Dissociation tandem mass spectrometry analysis was set with an isolation width of 2.0 Da. This was done above a threshold ion count of 10,000 in the MS survey scan with 30.0 s dynamic exclusion. The electrospray voltage was 2.0 kV. Automatic gain control was used to prevent overfilling of the ion trap, and 50,000 ions were accumulated for generation of MS/MS spectra. For MS scans, the *m*/*z* scan range was 350–1800, and for MS/MS scans was 200–2000.

### Peptides identification and relative quantitation

The resulting MS/MS data were analyzed using PEAKS Studio Software (8.5 Edition, Bioinformatics Solutions Inc., Waterloo, Canada). Tandem mass spectra were searched against the UniProt insect database (November 2024). Peptide spectral matches were validated based on q-values to 1% FDR (false discovery rate) using percolator. Trypsin was chosen as enzyme, and missed cleavages were allowed to two. Carbamidomethylation of cysteine residues (+57.02 Da) was set as fixed modification, deamidation (+0.98), oxidation (+15.99), hydroxylation (+15.99), light-labeled demethylation (+28.03) and medium-isotope labeled (+32.06) at lysine or N-terminus, were set as variable modifications. All other parameters were default settings, a fragment ion tolerance was set as 0.03 Da and a maximum precursor ion tolerance was 25 ppm. Dimethyl labeling-based quantitation method was applied to calculate the relative quantitation of light- and medium-labeled peptides at the MS level using PEAKS Q module of PEAKS Studio 8.5. Mass error tolerance was set as 15.0 ppm, and retention time range was set as 2.0 min. Bioinformatic analysis was performed using the OmicStudio tools at https://www.omicstudio.cn/tool.

### Validation of peptide biomarkers and application for distinguishing males from females

Based on fold change, p-value threshold settings, and volcano plot analysis, peptides significantly elevated in males relative to females were identified as potential peptide biomarkers. Female and male ES samples underwent digestion processing according to the protocol described in Sample preparation. UPLC–MS/MS was performed using a Shimadzu Nexera UPLC LC-20A system (Shimadzu, Kyoto, Japan) and a QTRAP 5500 Plus mass spectrometer (Applied Biosystems, Foster City, CA, United States) coupled with an ESI source operated in positive ion mode. An Acquity UPLC C_18_ column (2.1 mm × 100 mm, 1.7 μm) connected to an Acquity UPLC BEH Amide VanGuard pre-column (1.7 μm) was used for chromatographic separation. 0.1% FA in ACN (A) and 0.1% FA in water (B) composed the mobile phase. Samples (1 μL each) were separated using a gradient elution of 0–2 min with 10%–30% A, 2–6 min with 30%–50% A, and 6–7 min with 50%–50% A, 7–8 min with 50%–10% A, 8–9 min with 10%–10% A; at a flow rate of 0.3 mL min^−1^. The column was maintained at 35 °C. The eluate was directed to the mass spectrometer for analysis.

MS Analyst software version 1.5.2 was used to control the UPLC-MS/MS system. The ESI source parameters were set as: ion source temperature, 500 °C; ionization voltage, 5,500 V; desolvation temperature, 500 °C; curtain gas pressure, 40 psi; ion source gas 160 psi; ion source gas 260 psi. Peptide biomarker detection was performed in multiple-reaction monitoring (MRM) mode. The optimized collision energy (CE) and declustering potential (DP) values of selected Q1 and Q3 MRM transitions are summarized in Table 1.

## Results and discussion

### Strategy for peptide biomarkers discovery

The workflow of discovering ES peptide biomarkers proposed in this work is shown in Figure 1. Dimethyl labeling-based quantitative peptidomics analysis was implemented, trypsin-digested peptides from both male and female ES were identified and quantified. The methylation efficiency of N-terminal in light and medium samples was detected as 97.65% and 95.54%, respectively. By setting a filtering threshold, peptides with significantly higher abundance in males than in females were identified as potential peptide biomarkers. Furthermore, quantitative detection of selected peptide biomarkers was performed using UPLC-MS/MS in MRM mode as a targeted mass spectrometry approach, which also avoids suppression of other peptide signals. This strategy integrates as follows: (i) comprehensive identification of peptides from males and females; (ii) combining dimethyl labeling-based MS quantitative analysis with setting screening parameters, including fold change, p-value, trypsin digestion specificity, to find the peptide biomarkers in males; (iii) verifying peptide biomarkers through MRM mass spectrometry analysis. Proteomics analysis based on nanoLC Orbitrap MS provides comprehensive proteomic results. The relative abundance of selected biomarkers in male and female ES insects was detected via MRM mass spectrometry analysis. In multiple batches of *E. sinensis* samples. This method demonstrated good reproducibility in both the peak areas and retention times of the characteristic peptides (RSD < 10%). Its applicability across different instrument platforms has also been confirmed, as the peak shapes and retention times of the characteristic peptides were essentially consistent between SCIEX Triple Quad^TM^ 5500 and 6500 LC-MS/MS systems. Detailed results for each step will be presented in subsequent sections.

### Peptides identification and discovery of potential peptide biomarkers

Five batches of male ES insect samples were pooled, extracted and digested into peptides to prepare male ES samples; female ES samples were prepared using the same procedure. The peptides from male and female samples were dimethyl labelled with light and medium tags, respectively. Then dimethyl labeled male and female samples were pooled and quantified using nanoLC-MS/MS, as a result, 277 peptides were quantified as shown in Supplementary Table S1. Furthermore, in order to screen potential peptide biomarkers, the screening criteria and thresholds were set as follows: (i) peptides must exhibit tryptic-digestion characteristics; (ii) fold change >8 and p-value <0.01; (iii) identification confidence: −10lgP value ≥50, with high-quality MS/MS spectra. Through this screening, three peptide biomarkers were identified. Additionally, to normalize peptide relative abundance, a reference peptide was screened and selected. Conditions (i) and (iii) remained unchanged, while condition (ii) was set to a fold change between 0.95 and 1.05 with a p-value greater than 0.05. We compared FC > 5, >8, and >10 (p < 0.01) and selected FC >8 as the optimal balance, as FC >5 gave excessive false positives while FC >10 risked omitting true markers. All three peptides meeting FC >8 were validated by synthetic peptide-targeted MRM. One reference peptide was selected through this screening process. These four selected peptides were shown in Table 1.

EMSWIADTYAK (ES-Pep-1), IVPIVEPEVLPDGDHDLDR (ES-Pep-2) and TGAIVDVPVGDELLGR (ES-Pep-3) were finally selected as the peptide biomarkers of male ES. Peptide EAFSLFDKDGDGTITTK (ES-Pep-R) fit the filter conditions of internal reference, and was invariable in males and females. Thus, ES-Pep-R was selected as reference for normalizing ES-Pep-1, ES-Pep-2 and ES-Pep-3. The MS/MS spectra of ES-Pep-1, ES-Pep-2, ES-Pep-3 and ES-Pep-R were shown in Supplementary Figure S1. Taking the medium-isotopically labeled EMSWIADTYAK as an example, m/z 689.86 was identified as the precursor ion, with the N-terminal Glu and C-terminal Lys labeled with a medium isotope label (+32.06); the product y ions were identified as m/z 250.21 (y2), 413.27 (y3), 514.32 (y4), 629.34 (y5), 700.39 (y6), 813.47 (y7), 999.55 (y8), 1086.57 (y9), and 1217.63 (y10). Based on these MS/MS fragments, the amino acid sequences could be determined, and the other peptides could be determined in the same manner.

### Validation of peptide biomarkers to distinguish males from females

**FIGURE 2:**
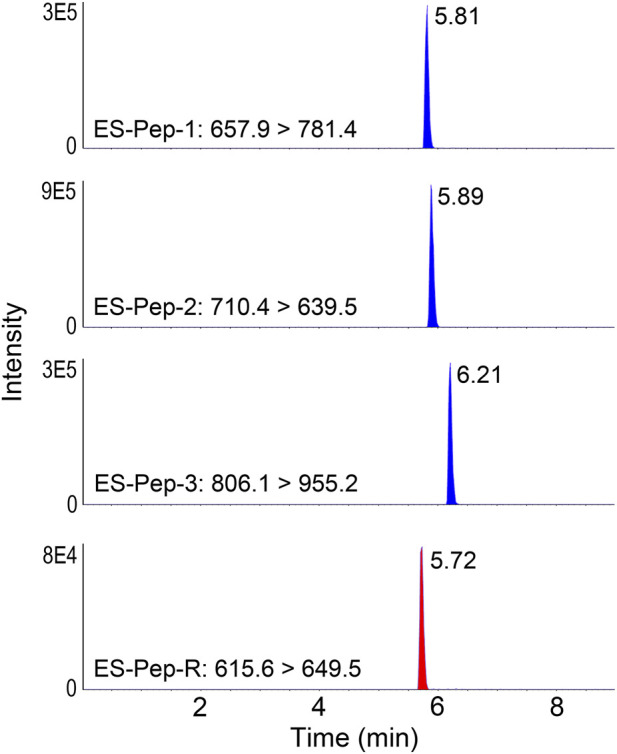
MRM chromatograms of ES-Pep-1, ES-Pep-2, ES-Pep-3 and ES-Pep-R.

To verify whether the peptide biomarkers were significantly higher in male insects than in females, three characteristic peptides and the internal reference peptide were synthesized. Detection conditions were optimized and shown in Table 1, the extracted ion chromatograms (XIC) were shown in Figure 2. The results demonstrated excellent response for all four peptides. To carry out relative quantifications of ES-Pep-1, ES-Pep-2 and ES-Pep-3 in males and females, these peptide biomarkers were analyzed by UPLC-MS/MS with ESI positive ionization in MRM mode. Compared to nanoLC-MS/MS, the MRM mode enables targeted detection of peptides while avoiding signal suppression caused by other peptides, thereby enhancing instrument sensitivity. The multi-charged ions ES-Pep-1 (carried 2+ charges), ES-Pep-2 (3+), ES-Pep-3 (2+) and ES-Pep-R (3+) were selected as the precursor ions generating the strongest Q1 signal. Under collision-induced dissociation (CID) conditions with optimized collision energy (CE), these precursor ions were fragmented into product ions, with the two strongest ions selected as Q3. Taking ES-Pep-1 as an example, its precursor ion was [M + 2H]^2+^ with an *m/z* value of 657.9 (2+). Following CID, it produced two fragment ions: 781.4^+^ (y8) and 668.2^+^ (y7), which were selected as product ions. Therefore, the MRM transitions 657.9 > 781.4 and 657.9 > 668.2 were selected.

**FIGURE 3:**
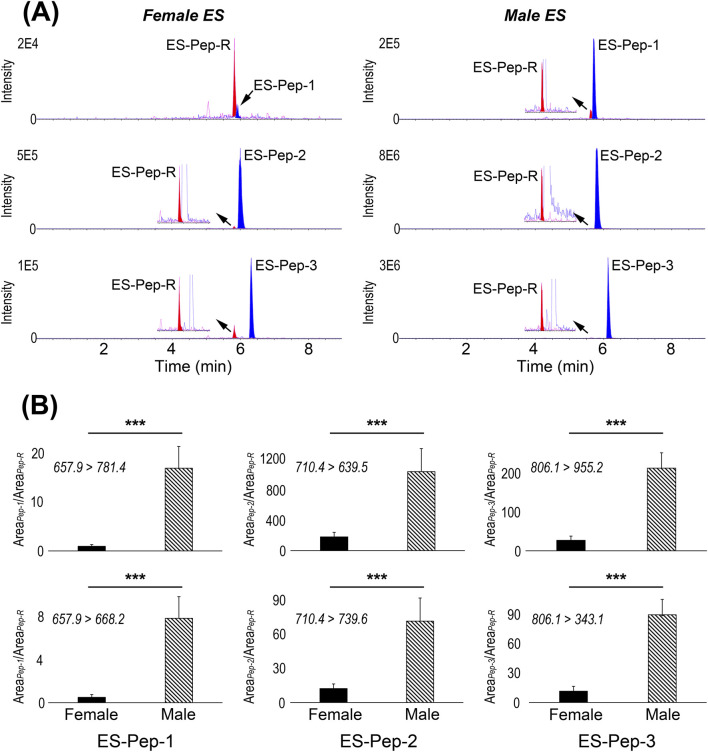
The levels of peptide biomarkers in male ES and female ES. **(A)** ES-Pep-1, ES-Pep-2, ES-Pep-3 and ES-Pep-R in male ES and female ES; **(B)** The relative abundance of ES-Pep-1, ES-Pep-2 and ES-Pep-3 in male ES and female ES.

The peak areas of ES-Pep-1, ES-Pep-2, ES-Pep-3 and ES-Pep-R in males and females (n = 7) were listed in Supplementary Table S2. The normalized peak area values for ES-Pep-1, ES-Pep-2, and ES-Pep-3 were calculated based on the ratios of ES-Pep-1, ES-Pep-2, and ES-Pep-3 to ES-Pep-R, respectively. As shown in Supplementary Table S3, the relative levels of ES-Pep-1 (657.9 > 781.4) ranged from 0.48 to 1.45 in females, and 12.54 to 23.52 in males; for ES-Pep-2 (710.4 > 639.5) ranged from 94.33 to 245.15 in females, and 671.57 to1455.00 in males; for ES-Pep-3 (806.1 > 955.2) ranged from 14.70 to 41.90 in females, and 157.75 to 281.45 in males. After normalization, the relative levels of these three peptides showed significant differences between females and males (All p-values were less than 0.001). Therefore, as shown in Table 2 and Figure 3, the results indicated that ES-Pep-1, ES-Pep-2 and ES-Pep-3 were reliable biomarkers for distinguishing males from females. Among these, ES-Pep-1 exhibited greatest differential expression, with the average relative level in males being 20 times that in females. This suggests that ES-Pep-1 may be the most suitable characteristic peptide among the three peptides. Consequently, it is suggested that the normalized peak area value of ES-Pep-1, ES-Pep-2 and ES-Pep-3 in females should not exceed 10.0, 537.2, and 126.2, respectively, which were the lowest detected content in males, adjusted downward by 20%. If the relative peak area of ES-Peps in the female ES samples exceed these values, which indicates that the sample is male ES or contains at least some male ES.

**TABLE 2:** Relative peak areas of characteristic peptides in female and male insects.

Peptides	Ion pairs	Relative peak areas (X ± sd, n = 7)		Average male/female ratio (X ± sd, n = 7)
		Female ES	Male ES	
ES-pep-1	657.9 > 781.4	0.95 ± 0.36	16.93 ± 4.45	20.00 ± 9.31
	657.9 > 668.2	0.49 ± 0.26	7.85 ± 2.02	21.18 ± 15.06
ES-pep-2	710.4 > 639.5	177.81 ± 59.12	1,027.58 ± 299.66	6.77 ± 4.29
	710.4 > 739.6	12.31 ± 4.07	71.88 ± 20.42	6.80 ± 4.21
ES-pep-3	806.1 > 955.2	27.27 ± 10.75	214.86 ± 39.94	9.28 ± 5.00
	806.1 > 343.1	11.55 ± 4.99	89.74 ± 15.73	9.35 ± 5.16

### Detection and quantitative analysis of simulated mixed male-female samples

Simulated mixed male-female ES samples with varying male concentrations were prepared to investigate the correlation between the relative abundance of peptide biomarkers and the percentage of male ES, assuming a constant ratio of peak areas between male and female ES biomarkers. Male and female ES were mixed at 90:10, 80:20, 60:40, 50:50, 40:60, 20:80 and 10:90 ratios by weight, then all the mixed ES samples were digested, and ES-Pep-1, ES-Pep-2, ES-Pep-3 and ES-Pep-R were analyzed by UPLC-MS/MS in MRM mode. The relationships between male ES percentages and normalized peak area value of ES-Pep-1, ES-Pep-2 and ES-Pep-3 were established, which are shown in Supplementary Figure S2 and Supplementary Table S3.

As shown in Supplementary Figure S2, the results indicate that in simulated mixed samples, the relative peak area values of ES-Pep-1, ES-Pep-2 and ES-Pep-3 exhibit a good linear relationship with the proportion of male insects, with correlation coefficients ranging from 0.9498 to 0.9957. Among these, the linear relationship between the relative peak area of the 710.4 > 639.5 ion pair of ES-Pep-2 and the proportion of male insects was the strongest (r^2^ = 0.9957), which suggested that ES-Pep-2 could serve as a potential peptide biomarker for detecting the proportion of adulterated males in female ES samples. Based on the relative contents of biomarkers, the proposed method can identify the authenticity of female ES samples and determine the adulteration ratio of males in female samples as well.

In the present work, three male ES derived characteristic peptides, ES-Pep-1, ES-Pep-2 and ES-Pep-3 were discovered and applied for distinguishing male ES from females. On the one hand, the LC-MS method developed can be used for rapid screening to distinguish between male and female specimens and detect adulteration for bulk purchasing of ES, thereby ensuring batch consistency and quality control. On the other hand, these three characteristic peptides can serve as quality markers during the processing of ES, allowing for the monitoring of how processes such as preparation and drying affect their stability. They also help establish quality standards based on the levels of these characteristic peptides, thereby enhancing quality control.

## Conclusion

In this study, a dimethyl labeling-based quantitative peptidomics analysis was developed for rapidly discovering peptide biomarkers for male ES from female ES. From those identified and quantified peptides, three peptides were selected as potential biomarkers for the distinguishing based on their relative contents. The adulteration ratio of males to females might be further calculated based on the ratios of peptide biomarkers’ relative contents. The presented work demonstrated high selectivity allowing rapid and accurate discrimination of male ES and had a superior application value for the in-depth authentication of insect-derived traditional Chinese medicines.

## Data Availability

The mass spectrometry proteomics data have been deposited to the ProteomeXchange Consortium via the PRIDE partner repository (https://www.ebi.ac.uk/pride/) with the dataset identifier PXD080333.

## References

[B1] Cai S. Zhao K. X. Jiang M. T. Han S. Y. Zheng Y. F. Liu X. (2021). Collagen derived species-specific peptides for distinguishing donkey-hide gelatin (asini corii colla). Chin. Herb. Med. 13, 261–266. 10.1016/j.chmed.2020.12.006 36117504 PMC9476752

[B2] Chinese Pharmacopoeia (2025). in *C. Pharmacopoeia* of the People’s Republic of China Vol. 1 (Beijing, China: China Medical Science Press).

[B3] Fan C. Xu Y. N. Li Y. F. Yang M. H. Han J. P. Pang X. H. (2024). DNA metabarcoding uncovers fungal communities in zingiberis rhizoma. Chin. Herb. Med. 16, 679–685. 10.1016/j.chmed.2023.12.001 39606262 PMC11589332

[B4] Feng G. Bi J. L. Jin W. F. Wang Q. Dan Z. K. Fan B. L. (2024). Effect of Rhei Radix et Rhizoma and Eupolyphaga Steleophaga on liver protection mechanism based on pharmacokinetics and metabonomics. Chin. Herb. Med. 16, 121–131. 10.1016/j.chmed.2023.10.002 38375045 PMC10874764

[B5] Fu X. Shao B. H. Wei X. Wang H. H. Chen X. Zhao T. T. (2022). Tubiechong:A review on ethnomedicinal uses, bioactive chemical constituents and pharmacological activities. J. Ethnopharmacol. 298, 115642. 10.1016/j.jep.2022.115642 35973633

[B6] Han S. Y. Zhao K. X. Cai S. Jiang M. T. Huang X. Z. Chen S. J. (2021). Discovery of peptide biomarkers by label-free peptidomics for discrimination of horn gelatin and hide gelatin from Cervus nippon temminck. Food Chem. 363, 130347. 10.1016/j.foodchem.2021.130347 34147893

[B7] Han D. Cheng Y. X. Yan Y. M. (2022). Pharmacological effects of eupolyphaga sinensis: a review. Mod. Chin. Med. 24, 2501–2513. 10.13313/j.issn.1673-4890.20220126003

[B8] He J. Y. Hu Q. P. Tong P. Z. Qiu Y. J. Li G. W. Chen X. D. (2023). Characteristic ion identification of eupolyphaga steleophaga and its preparations. Nat. Prod. Res. Dev. 35, 2108–2116+2126. 10.16333/j.1001-6880.2023.12.010

[B9] Hu L. J. Liu N. Li Y. C. Zhang Q. C. Cui X. B. Shan C. X. (2023). A novel strategy to identify the species-specific peptide biomarkers in Pheretima aspergillum (E. Perrier) based on enzymatic digestion followed by LC-MS/MS methods. J. Pharm. Biomed. Analysis 229, 115372. 10.1016/j.jpba.2023.115372 37018956

[B10] Li N. Yue B. B. Zhang J. H. Zhao Y. Jia J. M. (2015). Molecular identification of processed medicinal insects Chinese polyphaga based on cytb gene. China Pharm. 26, 4354–4356. 10.6039/j.issn.1001-0408.2015.31.12

[B11] Li Y. C. Hu L. J. Zhang Q. Liu R. Cui X. B. Chai C. (2022). Analysis of three peptide components in centipede by UFLC-MS/MS and its application in identification of centipede. J. Nanjing Univ. Chin. Med. 38, 945–952. 10.14148/j.issn.1672-0482.2022.0945

[B12] Liu R. Tang J. Y. Wu W. X. Zhao J. J. Zhu Z. Y. Gu H. O. (2023). Combination of mathematics and label‐free proteomics for discovering keratin‐derived specific peptide biomarkers to distinguish animal horn‐derived traditional Chinese medicines. J. Sep. Sci. 46, e2200949. 10.1002/jssc.202200949 36821105

[B13] Luo X. X. Bi Q. R. Huang D. D. Li Y. Yao C. L. Zhang J. Q. (2023). Characterization of natural peptides in pheretima by integrating proteogenomics and label-free peptidomics. J. Pharm. Analysis 13, 1070–1079. 10.1016/j.jpha.2023.06.006 37842652 PMC10568111

[B14] Ni Y. D. Zhu Y. Y. Xu L. X. Duan J. A. Xiao P. (2024). Pharmacological activities and mechanisms of proteins and peptides derived from traditional Chinese medicine. Sci. Traditional Chin. Med. 2, 260–275. 10.1097/st9.0000000000000054

[B15] Wang Y. Yan H. L. Wang Y. P. Yang H. L. Wei L. Xiao Y. (2012). Proteomics and transcriptome analysis coupled with pharmacological test reveals the diversity of anti-thrombosis proteins from the medicinal insect, Eupolyphaga sinensis. Insect Biochem. Mol. Biol. 42, 537–544. 10.1016/j.ibmb.2012.04.001 22727120

[B16] Wei X. Wang J. Deng Y. Y. Shao B. H. Zhang Z. F. Wang H. H. (2023). Tubiechong patching promotes tibia fracture healing in rats by regulating angiogenesis through the VEGF/ERK1/2 signaling pathway. J. Ethnopharmacol. 301, 115851. 10.1016/j.jep.2022.115851 36273748

[B17] Wu W. Ren Q. P. Li C. J. Wang Y. Y. Sang M. Zhang Y. (2013). Characterization and comparative profiling of MicroRNAs in a sexual dimorphism insect, Eupolyphaga sinensis walker. PLoS ONE 8, e59016. 10.1371/journal.pone.0059016 23620723 PMC3631196

[B18] Wu W. D. Fu M. F. Song H. W. Yue L. (2024). Fingerprint analysis of tubiechong. J. Traditional Chin. Veterinary Med. 43, 62–66. 10.13823/j.cnki.jtcvm.2024.043

[B19] Yang H. Shen Y. P. Xu Y. Maqueda A. S. Zheng J. Wu Q. (2015). A novel strategy for the discrimination of gelatinous Chinese medicines based on enzymatic digestion followed by nano-flow liquid chromatography in tandem with orbitrap mass spectrum detection. Int. J. Nanomedicine 10, 4947–4955. 10.2147/ijn.S82291 26345994 PMC4531023

[B20] Zhang Y. (2011). Authentication of Eupolyphaga sinensis and its counterfeits. Clin. J. Chin. Med. 24, 529+539.

[B21] Zhang N. Zhao Y. D. Shi Y. X. Chen R. Fu X. L. Zhao Y. X. (2019). Polypeptides extracted from Eupolyphaga sinensis walker *via* enzymic digestion alleviate UV radiation-induced skin photoaging. Biomed. and Pharmacother. 112, 108636. 10.1016/j.biopha.2019.108636 30802824

[B22] Zou H. Y. Liang Y. Y. Wu W. R. Jiang P. Y. Zhang Y. W. Lai H. L. (2025). Gender identification of eupolyphaga steleophaga based on mitochondrial genome differences of Eupolyphaga sinensis. Chin. Med. Mater. 12, 2993–2997. 10.13863/j.issn1001-4454.2025.12.009

