# Supplementary figures and images for: Discovery of peptide biomarkers in male Eupolyphaga sinensis Walker (Tubiechong) by dimethyl labeling-based quantitative peptidomics analysis
Source: Front Pharmacol. 2026 Jul 9;17:1799023. doi: 10.3389/fphar.2026.1799023 (PMC13392538; doi:10.3389/fphar.2026.1799023)

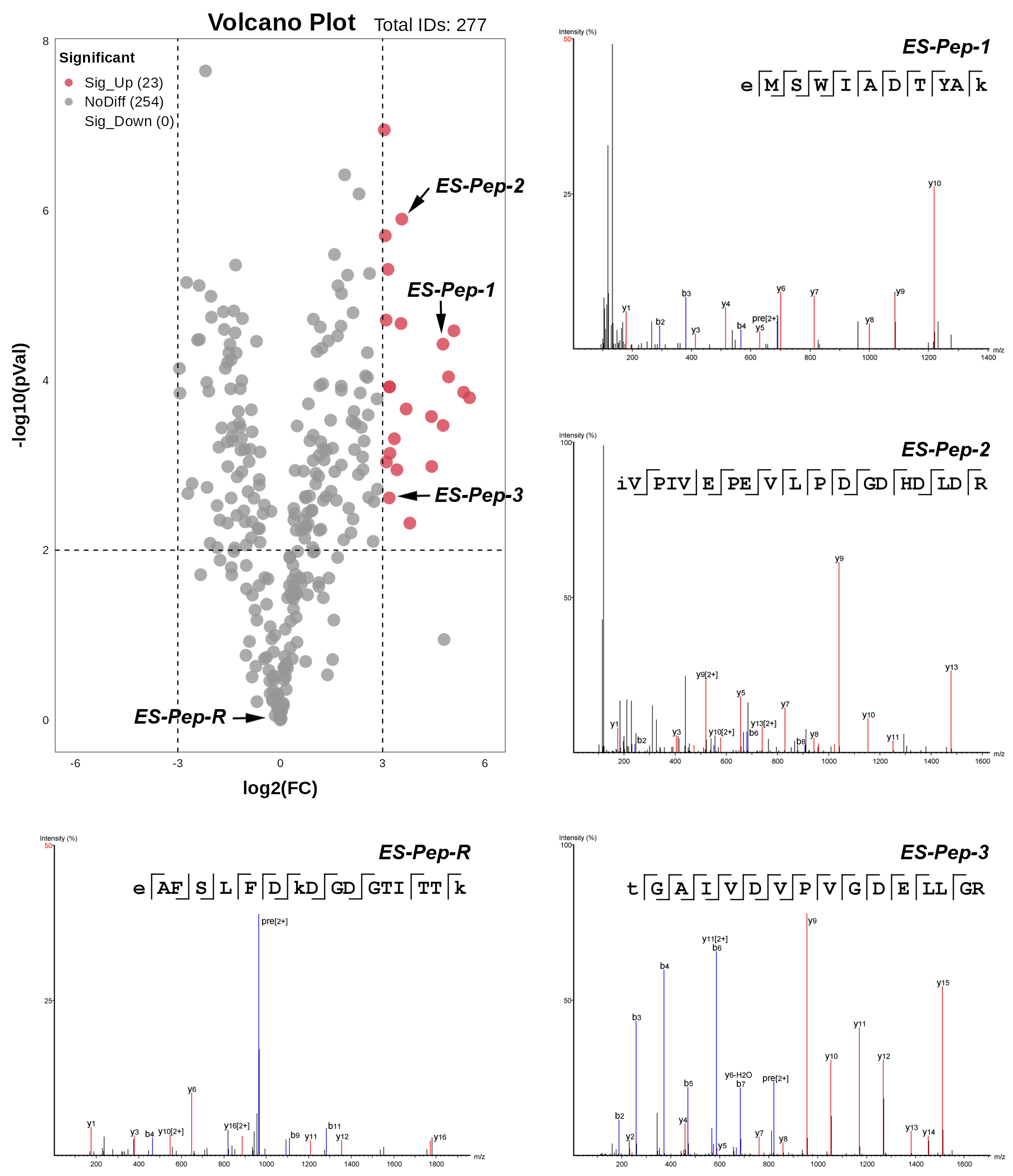

Supplement: Supplementary file 3 [file Image1.jpeg]

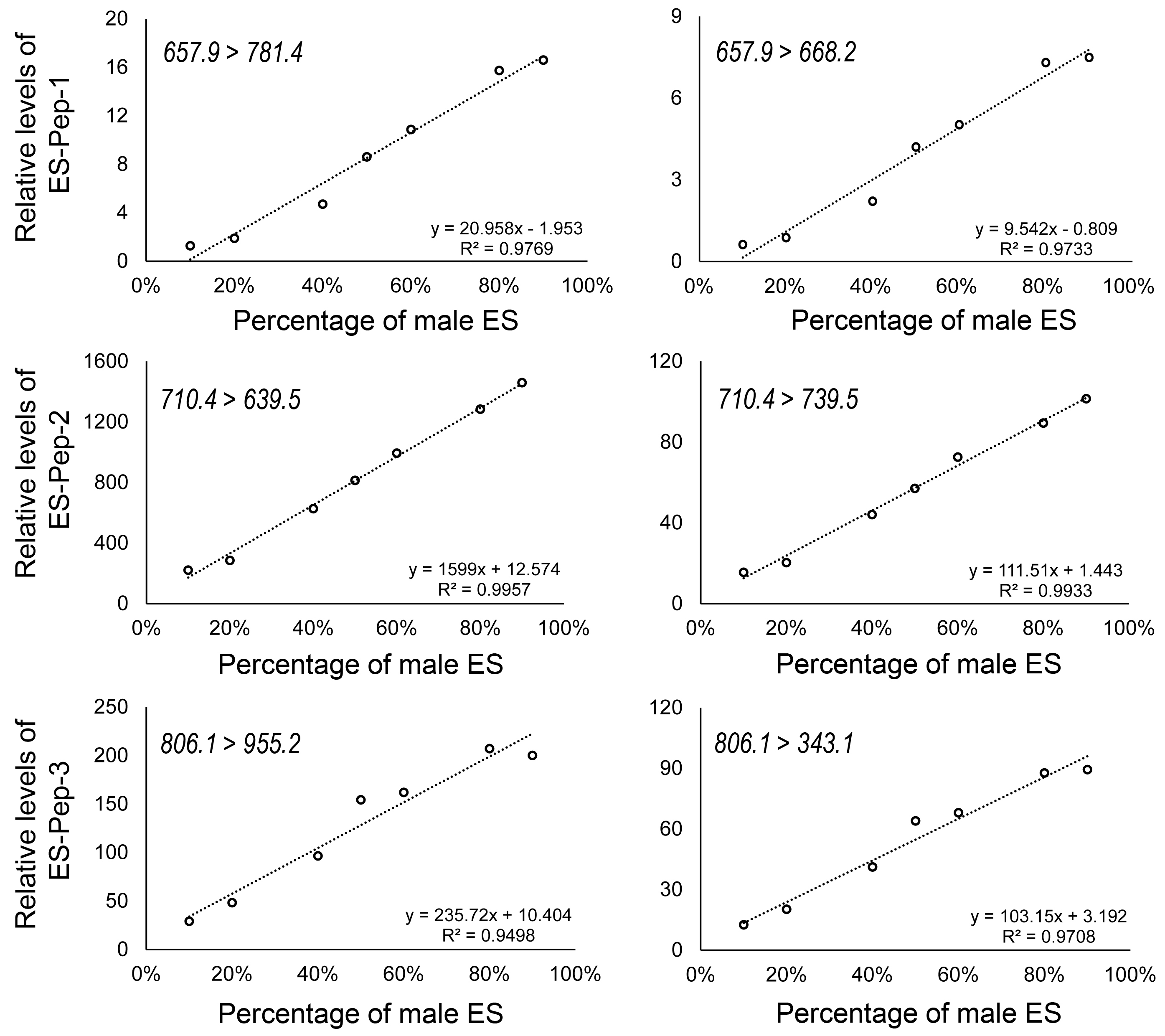

Supplement: Supplementary file 4 [file Image2.jpeg]
